# Endonuclease G restores lipid homeostasis in spontaneously hypertensive rats: implications for mitochondrial function

**DOI:** 10.3389/fphys.2026.1835167

**Published:** 2026-05-14

**Authors:** Petr Mlejnek, Miroslava Šimáková, Jan Šilhavý, František Liška, Tomáš Mráček, Josef Houštěk, Irena Marková, Martina Hüttl, Hana Malínská, Michal Pravenec

**Affiliations:** 1 Institute of Physiology, Czech Academy of Sciences, Prague, Czechia; 2 Institute of Biology and Medical Genetics, First Faculty of Medicine, Charles University and General University Hospital, Prague, Czechia; 3 Center for Experimental Medicine, Institute for Clinical and Experimental Medicine, Prague, Czechia

**Keywords:** endonuclease G (*Endog*) gene, insulin resistance, lipid and glucose metabolism, spontaneously hypertensive rat (SHR), transgenic

## Abstract

**Background:**

The mitochondrial endonuclease G (*Endog*) is best known for its roles in apoptosis and mitochondrial DNA maintenance, but accumulating evidence suggests broader metabolic functions. The spontaneously hypertensive rat (SHR) carries a natural loss-of-function mutation in *Endog* associated with mitochondrial dysfunction and left ventricular hypertrophy. We hypothesized that impaired mitochondrial oxidative metabolism, causing lipid dysregulation will be reversed by *Endog* restoration in the SHR.

**Methods:**

To determine the metabolic consequences of *Endog* restoration, we used two complementary rat models: (1) male SHR-*Endog* transgenic rats and (2) male SHR.BN-*Endog* congenic rats expressing wild-type *Endog*, each compared with their respective controls. We quantified adiposity, lipid and glucose homeostasis, liver and cardiac lipid accumulation, and white (WAT) and brown adipose tissue (BAT) metabolism.

**Results:**

*Endog* restoration in both transgenic and congenic rats led to reduced adiposity, lower serum triglycerides and non-esterified fatty acids (NEFA), and decreased ectopic lipid deposition in liver and heart. These improvements were accompanied by enhanced NEFA re-esterification in WAT, increased insulin-stimulated lipogenesis, and augmented palmitate oxidation in BAT. Systemic glucose and insulin levels were not significantly affected.

**Conclusion:**

Restoration of *Endog* expression improves lipid homeostasis and adipose tissue metabolic function in SHR. These findings suggest a role for *Endog* in the regulation of systemic lipid metabolism, with potential implications for mitochondrial-related metabolic processes.

## 1 Introduction

Mitochondria play a central role in the regulation of cellular and systemic metabolism by coordinating oxidative phosphorylation, fatty-acid oxidation, and redox balance. Impairments in mitochondrial function have been implicated in the pathogenesis of numerous cardiometabolic disorders, including dyslipidemia, insulin resistance, and nonalcoholic fatty liver disease. Although defects in mitochondrial bioenergetics are frequently observed in metabolic disease, the molecular determinants linking mitochondrial dysfunction to altered lipid metabolism remain incompletely understood.

Endonuclease G (*Endog*) is a nuclear-encoded mitochondrial nuclease that has traditionally been studied in the context of apoptosis (Cote et al., 1989; Cote and Ruiz-Carrillo, 1993). During apoptotic signaling, *Endog* is released from mitochondria and translocates to the nucleus, where it participates in chromosomal DNA degradation (Li et al., 2001). However, accumulating evidence suggests that *Endog* also performs important non-apoptotic functions within mitochondria. Previous studies have implicated *Endog* in mitochondrial DNA maintenance, regulation of mitochondrial biogenesis, and control of respiratory chain activity. These observations suggest that *Endog* may influence cellular metabolism through its effects on mitochondrial function (McDermott-Roe et al., 2011; Low, 2003; Wang et al., 2023).

The spontaneously hypertensive rat (SHR) carries a naturally occurring loss-of-function mutation in *Endog* that results in markedly reduced ENDOG protein expression and impaired mitochondrial efficiency. This mutation was previously identified as a determinant of cardiac hypertrophy and altered mitochondrial function in SHR (McDermott-Roe et al., 2011). In addition to cardiovascular abnormalities, SHR also exhibits multiple metabolic disturbances, including elevated circulating triglycerides, increased non-esterified fatty acids (NEFA), reduced oxidative capacity, and ectopic lipid accumulation in metabolically active tissues such as the liver and heart (Pravenec et al., 2014). These observations raise the possibility that *Endog* deficiency may contribute more broadly to systemic metabolic dysregulation. Despite increasing interest in the metabolic functions of *Endog*, its role in regulating systemic lipid metabolism remains poorly understood. In particular, it is not known whether *Endog* deficiency directly contributes to the dyslipidemia and abnormal lipid handling observed in SHR.

In the present study, we tested the hypothesis that impaired *Endog* function contributes to dysregulation of lipid metabolism in SHR and that restoration of wild-type *Endog* expression would normalize lipid handling and improve systemic metabolic homeostasis. To address this question, we used two complementary genetic strategies. First, we analyzed SHR.BN-*Endog* congenic rats carrying the wild-type Brown Norway *Endog* allele introgressed onto the SHR genetic background. Second, we studied SHR transgenic rats expressing wild-type *Endog*. Using these models, we examined the effects of *Endog* restoration on adiposity, circulating lipid levels, adipose tissue lipid metabolism, brown adipose tissue fatty-acid oxidation, and ectopic lipid accumulation in peripheral tissues. Together, these studies allow us to directly evaluate the role of *Endog* in linking mitochondrial function to systemic lipid metabolism in the SHR.

## 2 Materials and methods

### 2.1 Animals

**Figure 1:**
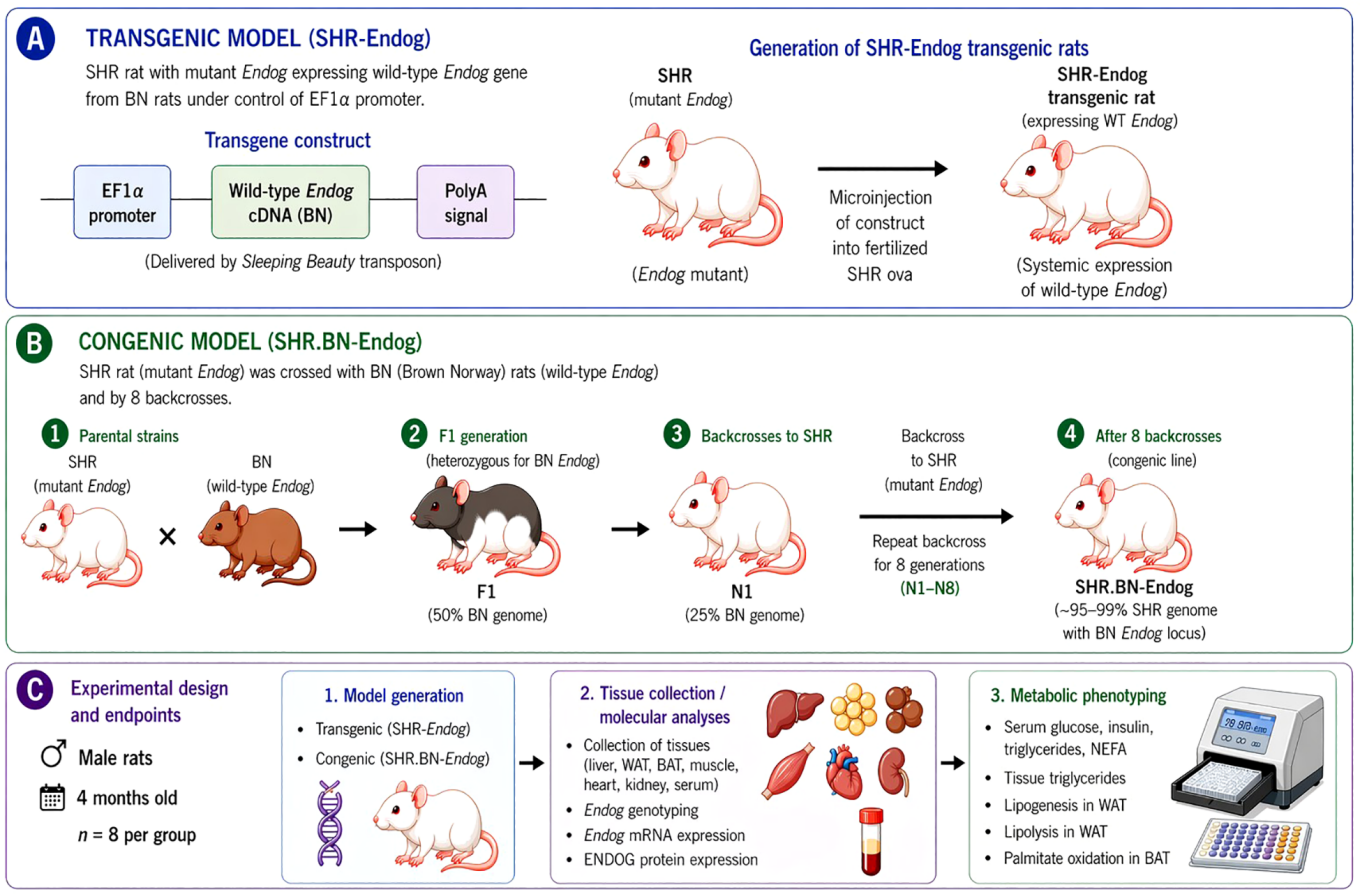
Generation of SHR-*Endog* transgenic and SHR.BN-*Endog* congenic rats and experimental workflow. **(A)** The SHR-*Endog* transgenic line was generated by introducing BN-derived wild-type *Endog* cDNA under the control of EF1α promoter into fertilized SHR ova using the Sleeping Beauty transposon system. **(B)** The congenic SHR.BN-*Endog* line was established by crossing SHR with BN rats carrying the wild-type *Endog* allele, followed by eight backcrosses to SHR (N1–N8), resulting in a predominantly SHR genetic background (98%) with the BN *Endog* locus retained. **(C)** Male rats (4 months old, n = 8 per group) were subjected to metabolic phenotyping and tissue analyses, including circulating metabolites, tissue triglyceride content, adipose tissue lipolysis and lipogenesis, brown adipose tissue fatty acid oxidation, and ENDOG expression.

To assess the functional consequences of the *Endog* mutation in the spontaneously hypertensive rat (SHR), we employed two complementary genetic approaches: we used the congenic SHR.BN-*Endog* line and the SHR-*Endog* transgenic line expressing wild-type *Endog* (**Figure 1**). These models provide distinct but synergistic advantages. The congenic line introduces the wild-type *Endog* allele from the normotensive Brown Norway (BN) rat into the SHR genetic background through targeted chromosome substitution. This approach preserves native gene regulation and physiological expression levels, providing a biologically relevant assessment of gene function. However, because the introgressed chromosomal segment may retain BN alleles from adjacent loci, phenotypic effects may be partially confounded by linked genes. To address this limitation, we generated the SHR-*Endog* transgenic line, which restores wild-type *Endog* expression in the SHR background without altering neighboring genomic regions. This transgenic model allows direct evaluation of *Endog* function and complements the congenic strategy.

Male rats from the SHR.BN-D3Rat108/D3Rat56 congenic subline carrying the wild-type BN *Endog* allele were compared with males from the SHR.BN-D3Rat108/D3Rat124 subline carrying a shorter (~8 Mbp) segment harboring the mutant SHR *Endog* allele. Congenic sublines were generated through eight backcrosses, and their congenic status was confirmed by genome-wide genotyping (McDermott-Roe et al., 2011). Four-month-old male rats (N = 8 per congenic subline) were studied.

The transgenic SHR/Ola-Tg(EF1a-*Endog*)136 strain (SHR-*Endog* transgenic) was generated by microinjecting fertilized SHR ova with a Sleeping Beauty construct containing BN-derived *Endog* cDNA under the control of the EF-1α promoter, together with mRNA encoding the SB100X transposase (Ivics et al., 2014). Transgenic animals were identified by PCR using primers *Endog*-F (5′-CGA CAC CTT CTA CCT GAG CA-3′) and *Endog*-R (5′-GGC CCT GTG CAG ACA TAA AC-3′). Four-month-old male rats (N = 8 per group) were studied.

All rats were bred and housed under standard conditions with unrestricted access to food and water. All procedures were performed in accordance with the Animal Protection Law of the Czech Republic and approved by the Ethics Committee of the Institute of Physiology, Czech Academy of Sciences, Prague.

### 2.2 Glucose utilization in isolated epididymal and brown adipose tissue

Distal portions of epididymal adipose tissue or interscapular brown adipose tissue were rapidly dissected and incubated for 2 h in Krebs–Ringer bicarbonate buffer containing 5 mmol/L glucose, 0.1 μCi/ml [¹^4^C]-U-glucose (UVVR, Prague), and 2% bovine serum albumin. Incubations were performed at 37 °C in sealed vials in a shaking water bath under 95% O_2_/5% CO_2_, with or without insulin (250 μU/ml). Incorporation of [¹^4^C]-glucose into neutral lipids was measured. Tissues were rinsed, extracted in chloroform:methanol (2:1), and lipids isolated at 4 °C overnight. After phase separation, aliquots were evaporated, reconstituted in scintillation fluid, and radioactivity was measured. Incremental glucose utilization was calculated as the difference between insulin-stimulated and basal incorporation.

### 2.3 Lipolysis in isolated epididymal adipose tissue

Basal and adrenaline-stimulated lipolysis were measured in epididymal adipose tissue incubated for 2 h at 37 °C in Krebs–Ringer phosphate buffer (pH 7.4) containing 3% bovine serum albumin, with or without adrenaline (0.25 µg/ml). Concentrations of NEFA and glycerol in the medium were quantified. Reesterification percentage was calculated using the formula (1 - [NEFA]/(3 × [glycerol])) × 100.

### 2.4 Palmitate oxidation in brown adipose tissue

Palmitate oxidation was assessed in interscapular brown adipose tissue incubated at 37 °C in Krebs–Ringer bicarbonate buffer containing 0.5 μCi/ml [¹^4^C]-U-palmitic acid complexed with bovine serum albumin (3 mg/ml) and 0.3 μmol/ml cold palmitate. After a 2-h incubation, 1.0 M hyamine hydroxide was injected into the central compartment of the incubation vessel, and 1 M H_2_SO_4_ was added to the main compartment to release CO_2_. Vessels were incubated for an additional 45 min. The hyamine hydroxide fraction was transferred to scintillation vials for radioactivity measurement.

### 2.5 Tissue triglyceride measurements

Liver, soleus muscle, and heart samples were powdered under liquid N_2_ and extracted for 16 h in chloroform:methanol. After adding 2% KH_2_PO_4_ and centrifugation, the organic phase was collected and evaporated under N_2_. The lipid residue was dissolved in isopropanol, and triglyceride content was measured enzymatically (Erba-Lachema, Brno).

### 2.6 Biochemical analyses

Blood glucose was measured by glucose oxidase assay (Pliva-Lachema) using tail-vein blood collected in 5% trichloroacetic acid and centrifuged. NEFA levels were determined using an acyl-CoA oxidase–based colorimetric kit (Roche Diagnostics, Mannheim, Germany). Serum triglycerides were measured by enzymatic assay (Erba-Lachema). Glycerol was quantified using a Sigma analytical kit. Serum insulin was measured using a rat insulin ELISA kit (Mercodia, Uppsala, Sweden).

### 2.7 Western blotting

Samples of fibroblasts or tissue homogenates were denatured at 56 °C for 15 min in a sample lysis buffer (2% (v/v) 2 mercaptoethanol, 4% (w/v) SDS, 50 mM Tris HCl, pH 7.0, 10% (v/v) glycerol, 0.017% (w/v) Coomassie Brilliant Blue R-250) and Tricine SDS-PAGE was performed on 10% (w/v) polyacrylamide slab gels. The gels were blotted onto a PVDF membrane (Immobilon P, Merck Millipore) by semidry electrotransfer at 0.8 mA/cm2 for 1 hour. Membranes were blocked in 5% non-fat dried milk dissolved in Tris buffered saline (TBS; 150 mM NaCl, 10 mM Tris HCl, pH 7.5) for 1 hour at room temperature. For detection of rat ENDOG protein, we used a rabbit monoclonal antibody (Abcam, ab76122; clone EP1665Y) at a dilution of 1:1000. To account for differences in mitochondrial content across samples, SDHA was used as a mitochondrial loading control, detected using a mouse monoclonal antibody (Abcam, ab14715) at a dilution of 1:10,000. For quantitative detection, the corresponding infra-red fluorescent secondary antibodies (Alexa Fluor 680, Life Technologies; IRDye 800, Rockland Immunochemicals) diluted in TBS supplemented with 0.1% (v/v) Tween-20 were used. The fluorescence was detected using ODYSSEY infra-red imaging system (LI-COR Biosciences) and the signal was quantified using Aida 3.21 Image Analyzer software.

### 2.8 Statistical analysis

Data are presented as means ± S.E.M. Differences between control and experimental groups were evaluated by paired or non-paired t tests as appropriate. P values < 0.05 were considered significant. For differences in gene expression we used the estimation statistics and graphics routines on the web application www.estimationstats.com to test for and display differences in group means (Ho et al., 2019; Claridge-Chang and Assam, 2016). Sample size (N = 8 per group) was determined based on our prior experience with similar metabolic phenotyping studies in SHR models. Although a formal *a priori* power calculation was not performed, the observed effect sizes for the main lipid-related parameters, including circulating triglycerides, NEFA levels, and adiposity, were large, supporting adequate power to detect biologically meaningful differences. This should nevertheless be considered a limitation for other measured parameters, particularly those with smaller effect sizes or greater variability.

## 3 Results

### 3.1 Derivation of SHR-*Endog* transgenic rats

**Figure 2:**
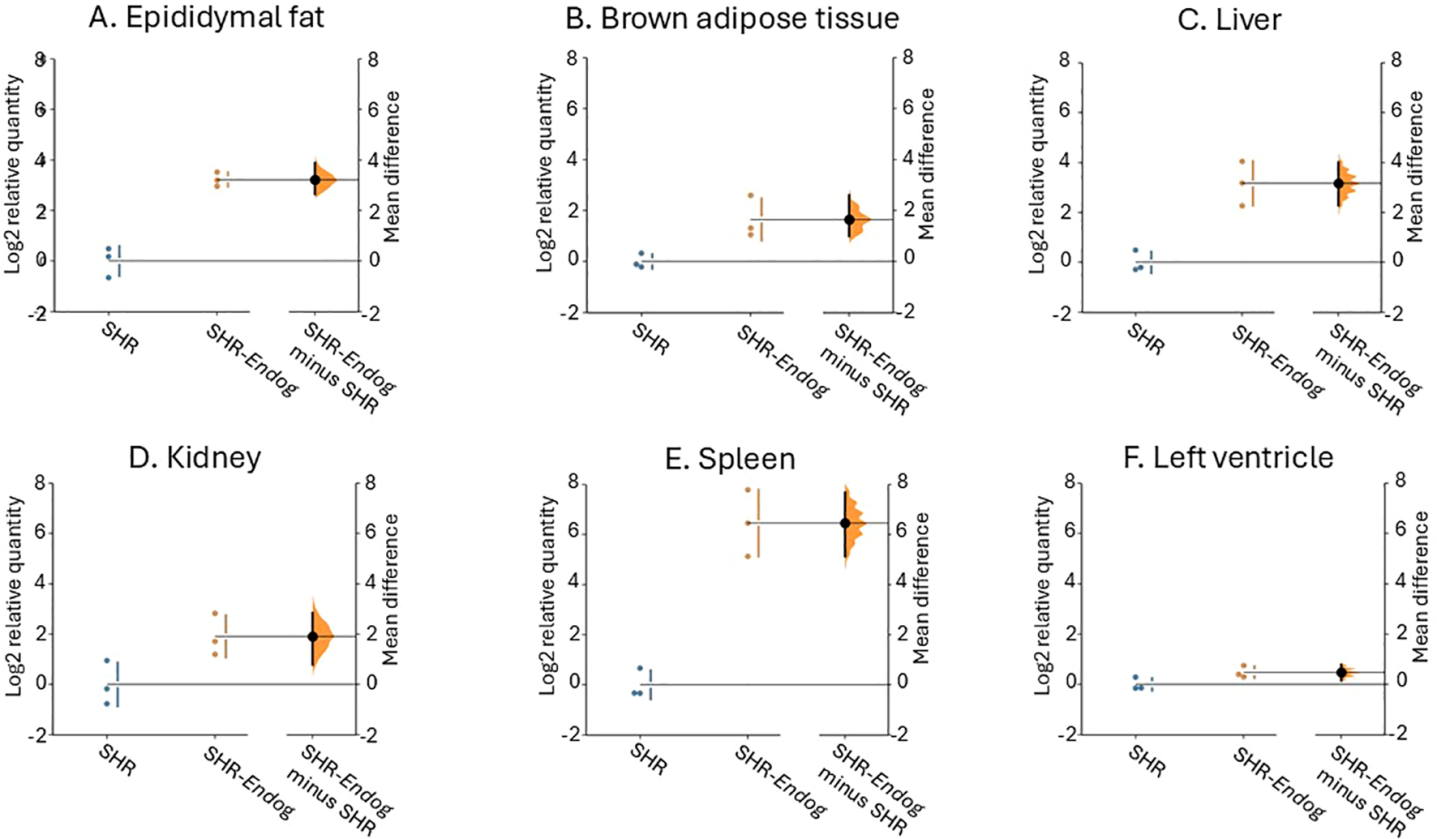
Tissue expression of *Endog* in SHR and SHR-*Endog* transgenic rats. The Gardner–Altman estimation plot (Ho et al., 2019) illustrates the difference in *Endog* expression between SHR and SHR-*Endog* transgenic rats across individual tissues. The left axis shows the raw data for each group, while the right floating axis displays the bootstrap sampling distribution of the mean difference between groups, shown as a dot with vertical error bars representing the 95% confidence interval. *Endog* expression was higher in SHR-*Endog* transgenic rats than in SHR controls in epididymal adipose tissue, brown adipose tissue, liver, kidney, spleen, and left ventricle **(A–F)**, consistent with systemic expression of the *Endog* transgene. Expression values are presented on a logarithmic scale (log-transformed relative expression), where the SHR group is centered at 0, and values >0 indicate increased *Endog* expression in SHR-*Endog* transgenic rats. Sample size was n = 3 animals per group.

**Figure 3:**
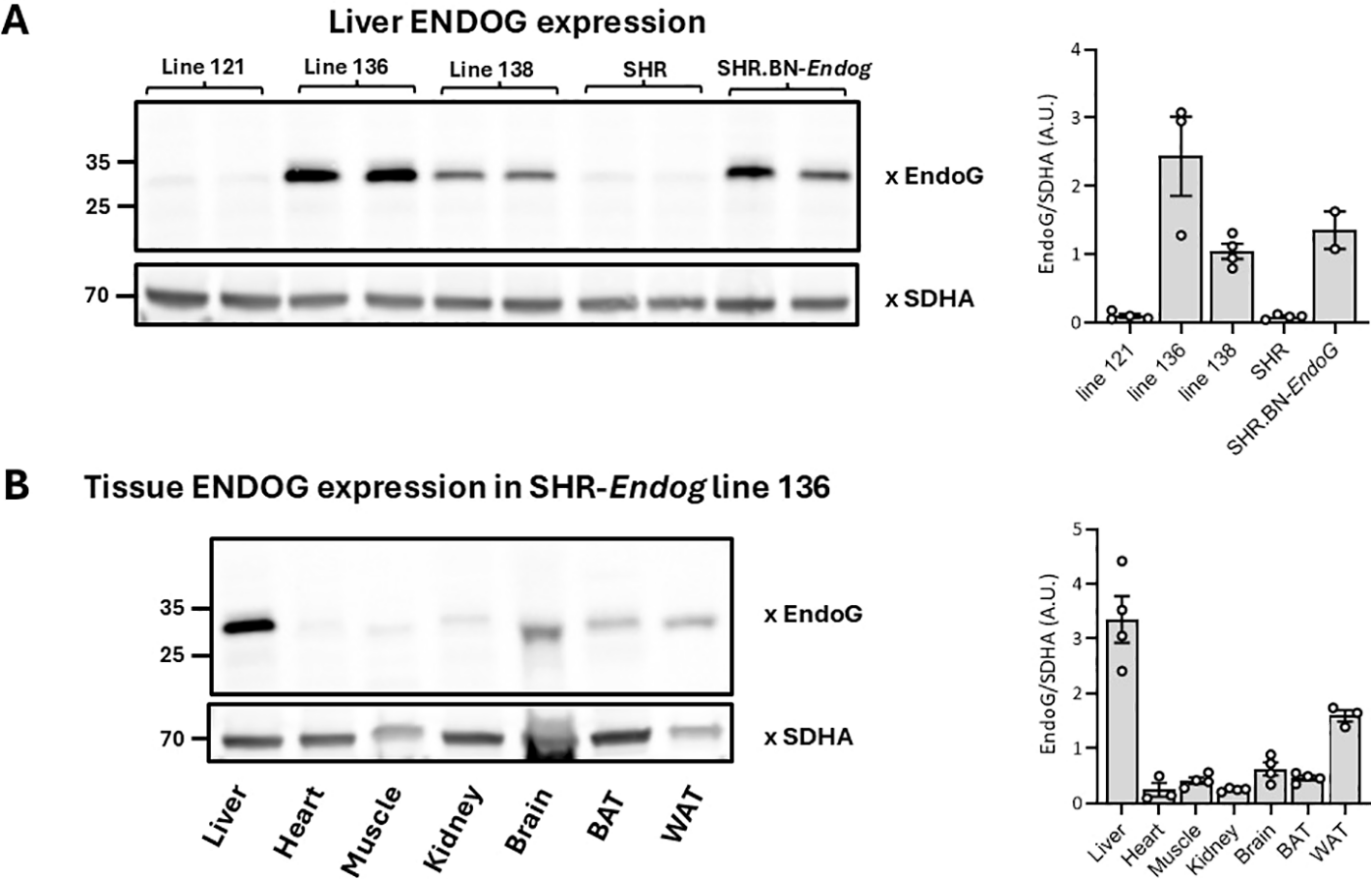
Rat ENDOG protein (~30 kDa) expression normalized to SDHA (succinate dehydrogenase complex, subunit A) in liver and selected tissues of SHR, SHR-*Endog* transgenic, and SHR.BN-*Endog* congenic rats. Representative Western blot analysis and corresponding densitometric quantification of ENDOG protein expression are shown. **(A)** The upper panel: Liver ENDOG expression in SHR-*Endog* transgenic lines 121, 136, and 138 compared with SHR controls and SHR.BN-*Endog* congenic rats. **(B)** The bottom panel: ENDOG protein expression across tissues [liver, heart, skeletal muscle, kidney, brain, brown adipose tissue (BAT), and white adipose tissue (WAT)] in SHR-*Endog* transgenic line 136. SDHA was used as a mitochondrial loading control to account for differences in mitochondrial content across samples. The bars correspond to the lanes shown in the representative blots.

Altogether 3 SHR-*Endog* transgenic founders were identified and line 136 was selected for further analyses based on significant transgene expression across tissues relevant for glucose and lipid metabolism. **Figure 2** shows expression of the transgene mRNA in SHR-*Endog* transgenic versus SHR rats. As can be seen, in SHR-*Endog* transgenic rats, there was significantly increased *Endog* wild-type mRNA in epididymal fat, liver and spleen which was paralleled by significant expression of ENDOG protein especially in the liver while no ENDOG expression was observed in SHR with mutant *Endog* variant (**Figure 3**).

### 3.2 *Endog* restoration reduces adiposity and improves lipid profiles

Both SHR-*Endog* transgenic and SHR.BN-*Endog* congenic rats exhibited reduced adiposity, as reflected by lower relative epididymal fat mass (−22% in transgenic, −32% in congenic rats). Circulating lipid parameters were also improved, with significantly lower serum triglycerides (−28% in transgenic, −13% in congenic rats) and NEFA levels (−32% in transgenic, −14% in congenic rats) compared to their respective controls. In contrast, serum glucose and insulin concentrations were not significantly different between groups (**Table 1**).

**Table 1:** Metabolic parameters in SHR.BN-*Endog*^SHR^ (with SHR mutant *Endog* variant) versus SHR.BN-*Endog^BN^* (with wild-type *Endog*) congenic sublines and SHR versus SHR-*Endog* transgenic rats.

Trait	SHR.BN-*Endog^SHR^* congenic subline	SHR.BN-*Endog^BN^* congenic subline	SHR	SHR-*Endog* transgenic
Body weight (g)	334 ± 3	324 ± 8	311 ± 5	302 ± 8
Relative epididymal fat weight (g/100 g body weight)	0.894 ± 0.022	0.673 ± 0.034**	0.985 ± 0.025	0.767 ± 0.031**
Relative weight of brown adipose tissue (g/100 g body weight)	0.092 ± 0.16	0.066 ± 0.07*	0.084 ± 0.002	0.068 ± 0.003**
Serum glucose (mmol/L)	6.7 ± 0.1	6.9 ± 0.1	7.3 ± 0.1	7.2 ± 0.1
Serum insulin (nmol/L)	0.220 ± 0.038	0.243 ± 0.037	0.245 ± 0.031	0.238 ± 0.053
Serum triglycerides (mmol/L)	0.63 ± 0.03	0.55 ± 0.02*	0.61 ± 0.05	0.44 ± 0.03*
Serum NEFA (mmol/L)	0.71 ± 0.03	0.61 ± 0.03*	0.60 ± 0.01	0.41 ± 0.02**
Muscle triglycerides (μmol/g)	1.33 ± 0.11	0.83 ± 0.14*	2.34 ± 0.33	2.00 ± 0.45
Heart triglycerides (μmol/g)	ND	ND	2.50 ± 0.26	1.32 ± 0.25**
Liver triglycerides (μmol/g)	3.92 ± 0.60	4.32 ± 0.60	10.85 ± 0.88	7.02 ± 0.59**
Basal lipolysis (NEFA) (μmol/g)	2.52 ± 0.30	2.03 ± 0.17	2.85 ± 0.28	1.63 ± 0.13*
Stimulated lipolysis (NEFA) (μmol/g)	5.69 ± 0.47	7.19 ± 0.54	4.51 ± 0.31	3.25 ± 0.40*
Palmitate incorporation in BAT (nmol palm./g/2h)	ND	ND	880 ± 33	1109 ± 38

* and ** denote P<0.05 and P<0.01, respectively; ND, not determined.

Analysis of tissue lipid content revealed reduced triglyceride accumulation in metabolically relevant tissues. In SHR-*Endog* transgenic rats, liver triglycerides were decreased by ~35% and heart triglycerides by ~47% compared to SHR controls, whereas muscle triglycerides were not significantly altered. In congenic rats, muscle triglycerides were significantly reduced (~38%), while liver triglycerides were not significantly changed (**Table 1**).

**Figure 4:**
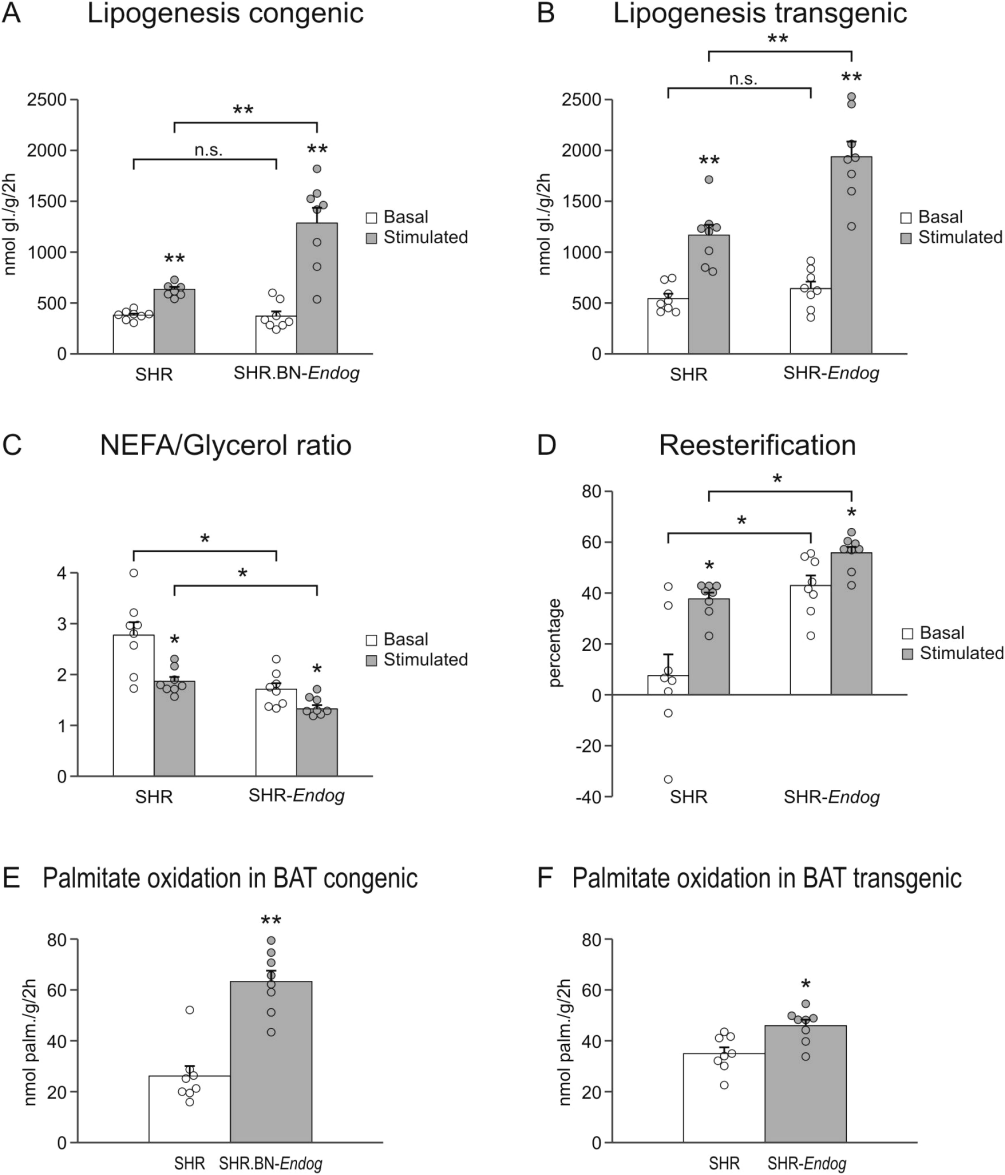
*Endog* increases adipose tissue fatty acid turnover and brown adipose tissue oxidative metabolism in SHR rats. In SHR.BN-*Endog* congenic **(A)** and SHR-*Endog* transgenic rats **(B)**, lipogenesis was increased under both basal and stimulated conditions. There was a lower NEFA/glycerol ratio **(C)** in SHR-*Endog* transgenic rats, consistent with enhanced fatty acid reesterification **(D)**. In brown adipose tissue of SHR.BN-*Endog* congenic **(E)** and SHR-*Endog* transgenic rats **(F)**, palmitate oxidation was significantly increased. Values represent mean ± SEM; individual data points are shown. Statistical analyses were performed using two-tailed unpaired Student’s *t*-test for comparisons between two groups. Sample size was *n* = 8 animals per group. *P < 0.01, **P < 0.001 versus SHR and SHR.BN-*Endog*^SHR^ subline, respectively. n.s., not significant.

Relative brown adipose tissue (BAT) weight was modestly reduced in both models (**Table 1**) despite increased oxidative capacity observed in functional assays (**Figure 4**). This indicates that *Endog* restoration enhances BAT metabolic activity rather than increasing BAT mass per se.

### 3.3 Enhanced fatty acid recycling and BAT oxidation following *Endog* restoration

The NEFA/glycerol ratio, an index of NEFA reesterification, was markedly reduced during lipolysis in SHR-*Endog* rats. Basal reesterification is ~5.6-fold higher in SHR-*Endog* compared with SHR, indicating markedly enhanced fatty acid recycling, consistent with improved intracellular lipid handling and potentially reduced lipotoxic NEFA spillover. The strain difference narrows under stimulation, suggesting *Endog* primarily enhances basal fatty-acid trapping, while stimulated lipolysis partially overrides this capacity. This was accompanied by increased insulin-stimulated lipogenesis in WAT (**Figure 4**) and significantly elevated palmitate oxidation in BAT of *Endog*-restored rats (**Figure 4**). In addition, SHR-*Endog* transgenic rats showed significantly increased palmitate incorporation into BAT (**Table 1**).

## 4 Discussion

### 4.1 *Endog* restoration improves lipid homeostasis

One of the most prominent findings of this study is the consistent improvement in lipid homeostasis observed in both *Endog* restoration models. SHR rats carrying the mutant *Endog* allele exhibit elevated circulating triglycerides and NEFA as well as increased lipid deposition in metabolically active tissues such as the liver and heart. These abnormalities were significantly attenuated following restoration of wild-type *Endog* (**Table 1**). Because both the transgenic and congenic models showed similar metabolic improvements, it is unlikely that these effects result from background genetic variation or linked loci. Instead, the results strongly support a direct role for *Endog* in regulating systemic lipid metabolism.

### 4.2 Enhanced fatty acid recycling in adipose tissue

Our data suggest that improved intracellular lipid handling in adipose tissue contributes to the reduction in circulating NEFA levels. During lipolysis, fatty acids released from triglycerides can either be exported into the circulation or recycled back into triglycerides through reesterification. In SHR-*Endog* rats, the NEFA-to-glycerol ratio during lipolysis was markedly reduced, indicating enhanced fatty acid reesterification (**Figure 4**). Increased recycling of fatty acids within adipocytes likely limits fatty acid spillover into the circulation and thereby reduces exposure of peripheral tissues to lipotoxic lipid intermediates. This mechanism is consistent with previous studies showing that impaired adipocyte lipid trapping contributes to systemic dyslipidemia and ectopic lipid deposition (Frayn, 1998).

### 4.3 Improved adipose tissue insulin responsiveness

*Endog* restoration also enhanced insulin-stimulated incorporation of glucose into lipids in white adipose tissue, indicating improved adipocyte insulin responsiveness (**Figure 4**). Notably, these changes occurred without detectable differences in circulating glucose or insulin levels (**Table 1**). Local improvements in adipocyte insulin sensitivity may therefore precede detectable systemic metabolic changes (Jensen, 2015; Shulman, 2014). Enhanced insulin-stimulated lipogenesis promotes triglyceride storage within adipocytes and further contributes to reduced circulating NEFA concentrations. Together, these findings suggest that *Endog* restoration improves both fatty acid recycling and insulin-regulated lipid storage in adipose tissue.

### 4.4 Increased fatty acid oxidation in brown adipose tissue

In addition to effects in white adipose tissue, *Endog* restoration increased palmitate oxidation in BAT (**Figure 4**). BAT plays an important role in lipid disposal through mitochondrial fatty acid oxidation and thermogenesis. Increased oxidative capacity in BAT may therefore contribute to the improved systemic lipid profile observed in *Endog*-restored rats. The increased fatty acid oxidation observed in BAT in our models is consistent with improved mitochondrial oxidative metabolism following restoration of *Endog* expression.

### 4.5 Differences between rat and mouse *Endog* models

Our findings differ from several studies using *Endog* knockout mice, which reported either reduced adiposity or protection from hepatic steatosis (Wang et al., 2023; Pardo et al., 2016). These discrepancies likely reflect differences in experimental design and biological context. In our study, *Endog* function was restored in a strain carrying a naturally occurring loss-of-function mutation, whereas mouse studies examined complete gene deletion in otherwise normal genetic backgrounds. The metabolic consequences of *Endog* deficiency may therefore depend on baseline mitochondrial function and the broader metabolic state of the organism. The SHR strain represents a genetic model characterized by hypertension and metabolic disturbances, conditions that may amplify the metabolic effects of *Endog* deficiency. Recent evidence further supports a role of *Endog* in mitochondrial metabolic regulation. Gao et al. (2023) showed that *Endog* acts downstream of *Sirt6* to enhance mitochondrial biogenesis and antioxidant defense, facilitating adaptation to increased fatty acid flux. This is consistent with our findings that *Endog* restoration increases palmitate oxidation in brown adipose tissue and improves lipid handling, supporting a role for *Endog* in promoting efficient fatty acid utilization and limiting lipotoxicity.

### 4.6 *Endog* as a link between mitochondrial function and lipid metabolism

*Endog* has previously been implicated in mitochondrial DNA maintenance, mitochondrial biogenesis, and regulation of respiratory chain activity. Impaired mitochondrial function can profoundly affect cellular lipid metabolism by limiting fatty acid oxidation and altering intracellular lipid flux. The improvements in adipose tissue lipid handling and BAT oxidation observed in *Endog*-restored rats are consistent with improved mitochondrial metabolic capacity. Although further studies will be required to define the precise molecular mechanisms involved, our results support a model in which *Endog* contributes to the maintenance of mitochondrial function and thereby influences systemic lipid metabolism.

## 5 Conclusions

In summary, restoration of wild-type *Endog* expression in spontaneously hypertensive rats improves lipid homeostasis, enhances adipose tissue lipid handling, and reduces ectopic lipid accumulation in metabolically important tissues. These findings identify *Endog* as a regulator of systemic lipid metabolism and suggest that impaired *Endog* function may contribute to metabolic disturbances associated with cardiometabolic disease. Further studies will be required to determine how *Endog* modulates mitochondrial metabolism and whether similar mechanisms operate in human metabolic disorders.

## 6 Study limitation

A limitation of the present study is that only 4-month-old male rats were examined, which may limit the generalizability of our findings. Sex- and age-dependent differences in lipid metabolism and mitochondrial function are well recognized, and it is therefore possible that the metabolic effects of *Endog* may differ in females or at other stages of development. Future studies will be required to determine whether the observed effects of *Endog* on lipid homeostasis are influenced by sex or age.

## Data Availability

The original contributions presented in the study are included in the article. Further inquiries can be directed to the corresponding author.
